# Status of pyrethroid resistance in *Anopheles gambiae s. s.* M form prior to the scaling up of Long Lasting Insecticidal Nets (LLINs) in Adzopé, Eastern Côte d’Ivoire

**DOI:** 10.1186/1756-3305-5-289

**Published:** 2012-12-11

**Authors:** Ludovic P Ahoua Alou, Alphonsine A Koffi, Maurice A Adja, Serge B Assi, Philippe K Kouassi, Raphael N’Guessan

**Affiliations:** 1 Institut Pierre Richet (IPR), BP 47, Abidjan, Côte d’Ivoire; 2 Laboratoire de Zoologie et Biologie Animale, Université Felix Houphouët-Boigny de Cocody, 22 BP 582, Abidjan 22, Côte d’Ivoire; 3 London School of Hygiene and Tropical Medicine, Keppel Street, London, WC1E 7HT, UK; 4 Centre de Recherche Entomologique de Cotonou, Cotonou, 06 BP 2604, Bénin

## Abstract

**Background:**

The growing development of pyrethroid resistance constitutes a serious threat to malaria control programmes and if measures are not taken in time, resistance may compromise control efforts in the foreseeable future. Prior to Long Lasting Insecticidal Nets (LLINs) distribution in Eastern Cote d’Ivoire, we conducted bioassays to inform the National Malaria Control Programme of the resistance status of the main malaria vector, *Anopheles gambiae s. s.* and the need for close surveillance of resistance.

**Methods:**

Larvae of *An. gambiae s. s.* were collected in two areas of Adzopé (Port-Bouët and Tsassodji) and reared to adults. WHO susceptibility tests with impregnated filter papers were carried out to detect resistance to three pyrethroids commonly used to develop LLINs: permethrin 1%, deltamethrin 0.05% and lambda-cyhalothrin 0.05%. Molecular assays were conducted to detect M and S forms and the L1014F *kdr* allele in individual mosquitoes.

**Results:**

Resistance, at various degrees was detected in both areas of Adzopé. Overall, populations of *An. gambiae* at both sites surveyed showed equivalent frequency of the L1014F *kdr* allele (0.67) but for all tested pyrethroids, there were significantly higher survival rates for mosquitoes from Tsassodji (32–58%) than those from Port-Bouët (3–32%) (p < 0.001), indicating the implication of resistance mechanisms other than *kdr* alone. During the survey period (May–June) in this forested area of Côte d’Ivoire, *An. gambiae s. s.* found were exclusively of the M form and were apparently selected for pyrethroid resistance through agricultural and household usage of insecticides.

**Conclusion:**

Prior to LLINs scaling up in Eastern Côte d’Ivoire, resistance was largely present at various levels in *An. gambiae*. Underlying mechanisms included the high frequency of the L1014F *kdr* mutation and other unidentified components, probably metabolic detoxifiers. Their impact on the efficacy of the planned strategy (LLINs) in the area should be investigated alongside careful monitoring of the trend in that resistance over time. The need for alternative insecticides to supplement or replace pyrethroids on nets must be stressed.

## Background

Malaria vector control strategies rely heavily upon the use of insecticide treated nets (ITNs) and indoor residual spraying (IRS). Pyrethroids are the most commonly used insecticides for IRS and also are the only compounds currently approved by the World Health Organization Pesticide Evaluation Scheme (WHOPES) for ITNs [1]. Unfortunately, the growing development of pyrethroid resistance threatens to undermine malaria control programmes [2]. Although the epidemiological significance of pyrethroid resistance has yet to be demonstrated [3], there are indications, at least in experimental huts and rural households, that ITNs are losing their protective power [4,5].

A well characterized mechanism of pyrethroid resistance in the malaria vector *Anopheles gambiae* is pyrethroids target site insensitivity in the voltage-gated sodium channel that induces knockdown resistance (*kdr*) [6,7]. In *An. gambiae s. s.* two alternative amino acid substitutions at the same position (L1014F and L1014S) confer resistance. The first mutation, involving a leucine-to-phenylalanine substitution originally found in West Africa is commonly termed L1014F *kdr* (*kdr-w*) [6], whereas the latter mutation found in East Africa and characterized by a serine substitution at the same position is termed L1014S *kdr* (*kdr-e*) [7]. First detected among *An*. *gambiae* field populations from Côte d’Ivoire and Burkina-Faso [6], the L1014F*kdr* is now widespread across West Africa and in some parts of Central and Eastern Africa [3,8,9]. The L1014F *kdr* mutation was initially detected in the S molecular form of *An*. *gambiae s. s.*[10] but has now been reported in both S and M forms from West and Central Africa [8,11,12].

In Côte d’Ivoire, the National Malaria Control Programme (NMCP) strategies are based on effective case management and high coverage of populations with Long Lasting Insecticidal nets (LLINs), particularly for children <5 yrs old and pregnant women. Aided by the Global Fund Initiatives (Round 6), the NMCP implemented in 2010 a free mass distribution of LLINs aimed at reducing malaria morbidity and mortality. However, several studies have demonstrated an increase in the frequency of the knockdown resistance gene mutations in *An. gambiae s. s.* following a nationwide LLINs implementation [13-15]. Increasing resistance in malaria vectors may have important implications for vector control programmes, mainly when these are based on the scaling up of LLINs or IRS.

Adzopé, a district hyper-endemic for malaria [16] is one of 19 health districts in Côte d’Ivoire targeted for the distribution of LLINs. Malaria vector susceptibility/resistance status to commonly used insecticides is well documented across the country (North-south-West) where LLINs are being scaled up, except the Eastern district of Adzopé.

This paper therefore reports, prior to LLINs distribution, the pyrethroid susceptibility/resistance status and the prevalence of the L1014F *kdr* allele in the main malaria vector, *An. gambiae s. s.* found in the area. The study would inform the NMCP of the resistance status of the main malaria vector, *An. gambiae s. s.* in the area and the need for its close surveillance.

## Methods

### Study sites

The study was conducted at Adzopé (6°10 N, 3°85 W) a forested district in Eastern Côte d’Ivoire.

**Figure 1:**
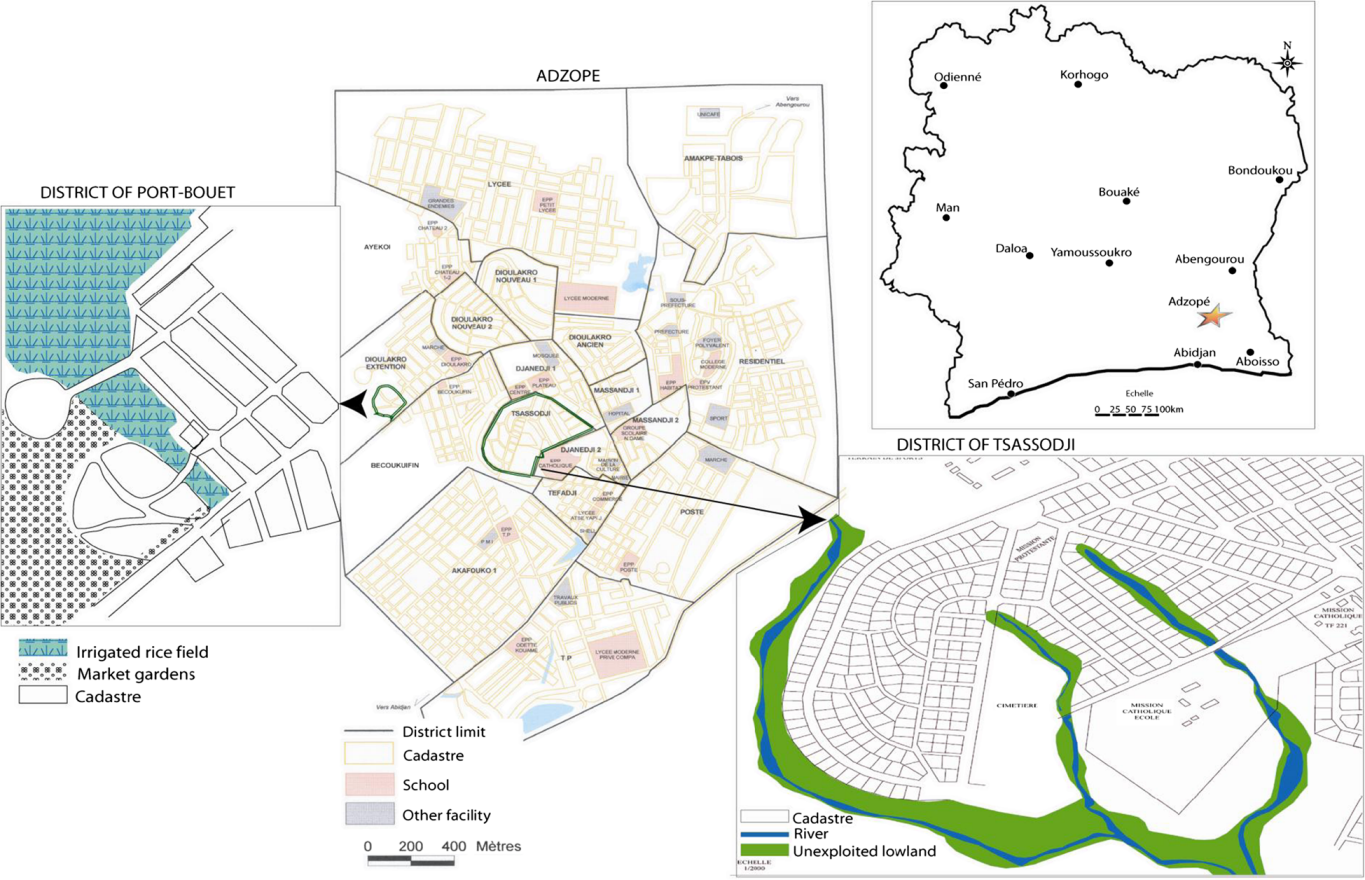
Map of the mosquito collection sites in the Adzopé city.

Two sampling sites in the district were chosen owing to their different history of pesticide usage and ecology. The sampling sites were (Figure 1):

(i) Tsassodji, located in the heart of Adzopé city, with no farming practices causing water retention, and;

(ii) Port-Bouët, on the outskirts of the city, with irrigated vegetable production and rice field.

Larvae from Tsassodji were mainly collected from temporary breeding sites such as puddles, shallow wells, gutters, footprints, tyre tracks maintained by rainfall during the collection period. By contrast, at Port-Bouët, the larvae were sampled in rice and vegetable areas.

### Pyrethroid susceptibility tests

During the raining season, between May and June 2007, *An. gambiae* larvae were collected from the breeding sites at each location and reared to the adult stage. Four batches of 20–25 unfed female mosquitoes, 2–3 day-old, were exposed to diagnostic concentrations of permethrin 25/75 (1%), deltamethrin (0.05%) and lambda-cyhalothrin (0.05%) filter papers using WHO standard cylinder kits [17]. Mosquitoes exposed to untreated filter papers served as controls. The number of mosquitoes knocked down at regular intervals during the exposure period was scored and time to knock down 50% and 95% of the exposed mosquitoes (KDT_50_ and KDT_95_) were determined. At the end of the exposure period, mosquitoes were transferred to holding tubes and given access to sugar food (10% solution). Mortality was scored 24 h after the holding period. A laboratory susceptible *An. gambiae* strain Kisumu was used as a reference. Abbott’s formula was used to correct the observed mortality when the rates were between 5% and 20%. All specimens were individually kept on silica gel in Eppendorf tubes for molecular analysis.

### Molecular assays

Genomic DNA was extracted from individual mosquitoes according to Collins *et al.*[18] and used for polymerase chain reaction (PCR) analysis. *Anopheles gambiae s. l.* were identified to species according to Scott *et al.*[19] then *An. gambiae s. s.* to molecular forms according to Favia *et al.*[20]. Both live and dead specimens from the bioassay tests were assayed using allele-specific PCR for the detection of the *kdr* L1014F mutation as per Martinez-Torres *et al.*[6]. For each site, the PCR was run on an equal number of mosquito samples, randomly selected among the live and dead individuals after the exposure to the pyrethroid-treated papers.

### Data analysis

The WHO criteria for discriminating individuals for susceptibility/resistance status were applied: 98–100% mortality indicating susceptibility; 80–97% mortality requiring confirmation of resistance; and <80% mortality suggesting resistance [17]. Knockdown data were analyzed using the PoloPlus 1.0 software (LeOra Software). KDT_50_ and KDT_95_ were generated by means of a log-time probit model. Genotype frequencies at the L1014F *kdr* locus were compared to Hardy-Weinberg expectations by using the exact probability test developed in Genepop 4.0 software [21]. The genotypic differentiation of the L1014F *kdr* locus in *An. gambiae s. s.* populations from the two sites was also tested using the Fischer exact test with the same software.

## Results

### Bioassays

Table 1 shows the insecticide susceptibility/resistance status of wild *An. gambiae* from Adzopé, relative to the Kisumu strain. The mortality rates in the control never exceeded 5% and so there was no need to correct with Abbott’s formula. All pyrethroid-treated papers killed 100% of susceptible *An. gambiae* Kisumu, indicating the accuracy of the impregnation and good bio-availability of the pyrethroids active ingredients on the papers. Generally, mortality rates with all pyrethroids were significantly higher with the Port-Bouët populations (68–97%) than with Tsassodji area (42–68%) (p < 0.001). Mortality rate to permethrin amongst population from Port-Bouët was <69%, indicating resistance. However, this population showed higher vulnerability to deltamethrin (96.7% mortality) and lambda-cyhalothrin (84.3%) compared to permethrin. Mosquitoes from Tsassodji were resistant to all three pyrethroids tested, with mortality rates <68%.

**Table 1:** **Knockdown times (KDTs) and mortality rates of *Anopheles gambiae *M form after exposure to diagnostic concentrations of pyrethroids on filter papers**

**Insecticide**	**Mosquito population**	**N**	**Knockdown effect**						**Mortality (%)**	**Status**
			**Knockdown time**				**KDT**_**50**_**Ratio**			
			**KDT**_**50**_**(min)**	**CL 95%**	**KDT**_**95**_**(min)**	**CL 95%**	**RR**_**50**_	**CL 95%**		
Permethrin 1%	Kisumu	96	9.7	9.1–10.2	13.7	12.7–15.4	-		100	S
	Port-Bouët	95	63.4	54.5–81.0	192.8	131.7–391.9	6.5	5.6–7.6	68.4^b^	R
	Tsassodji	100	76.2	64.7–96.6	329.8	219.6–618.7	7.9	6.4–9.6	42.0^a^	R
Deltamethrin 0.05%	Kisumu	96	21.2	18.1–24.5	38.0	31.5–53.4	-		100	S
	Port-Bouët	92	33.8	28.8–39.3	73.8	58.8–111.7	1.6	1.5–1.7	96.7^b^	SR
	Tsassodji	102	50.4	46.7–55.2	133.7	110.5–174.0	2.4	2.2–2.6	58.8^a^	R
Lambda-cyhalothrin 0.05%	Kisumu	95	27.5	24.5–30.3	43.4	38.4–52.9	-		100	S
	Port-Bouët	89	44.7	41.3–48.7	82.6	70.8–106.7	1.6	1.5–1.8	84.3^b^	SR
	Tsassodji	102	55.9	52.1–61.4	125.6	104.4–164.8	2.0	1.8–2.2	67.7^a^	R

N: Total number of mosquitoes exposed to each insecticides; KDT_50_ and KDT_95_: Knockdown time (minutes) for 50% and 95% of mosquitoes; CL 95%: 95% confidence limits; RR_50_: Resistance ratio at Kd_50_ level (KDT_50_ of wild population / KDT_50_ of susceptible strain); Mortality (%): mortality rate 24 h post-exposure; S indicates susceptibility; SR indicates suspicion of resistance that needs to be confirmed; R suggests resistance.

For each insecticide, numbers with different superscript differ significantly at 5% level.

KDT_50_s for all field populations (Tsassodji and Port-Bouët) increased significantly compared to the baseline susceptible strain Kisumu. With both mosquito populations, KDT_50_s were significantly higher for permethrin (>60 min) than for deltamethrin (33.8–50.4 min) and lambda-cyhalothrin (44.7–55.9 min). The Resistance Ratios (RR_50_s) as assessed by the ratios of the knock down times were moderate with permethrin (6.5–7.9-fold) to low with deltamethrin and lambda-cyhalothrin (1.6–2.4-fold). Clearly, deltamethrin and lambda-cyhalothrin showed greater toxicity than permethrin to both *An. gambiae* populations. The highest levels of resistance to pyrethroids were observed among populations from the Tsassodji area.

### Molecular assays

All *An. gambiae s. l.* from Adzopé tested were identified as *An. gambiae s. s.* and of the M form (Table 2). An attempt was made to establish the relationship between the expression of the L1014F *kdr* alleles and survivorship of mosquitoes in the bioassay data. It appeared that all three genotypes (SS, RS and RR) were observed in survivors as well as in dead bodies in both populations of *An. gambiae s. s.*, although at markedly different frequencies. In Port-Bouët, the 1014 F allele frequency was similar between both sub-groups (0.77 in survivor group versus 0.57 in dead group; χ^2^ = 5.30; df = 2; p = 0.07). The trend among the population from Tsassodji was similar to Port-Bouët, with no significant difference in *kdr* allele expression between survivors and dead samples of mosquitoes (0.71 versus 0.63; χ^2^ = 1.04; df = 2; p = 0.59).

**Table 2:** **Distribution of the *kdr-w *allele in *Anopheles gambiae *M form from Port-Bouët and Tsassodji**

**Mosquito sample**	**Phenotype**	**N**	**L1014F *kdr *mutation**			**F(1014 F)**	**P(HW)**
			**SS**	**RS**	**RR**		
Port-Bouët	Alive	28	1	11	16	0.77	
	Dead	28	8	8	12	0.57	
	Total	56	9	19	28	**0.67**	0.13
Tsassodji	Alive	19	1	9	9	0.71	
	Dead	15	2	7	6	0.63	
	Total	34	3	16	15	**0.68**	1.00
Total tested		90	12	35	43	**0.67**	0.34

N: Number of tested mosquito; S = Susceptible allele; R = Resistance allele; F(*kdr*) = L1014F *kdr* allelic frequency; P(HW) = Goodness of fit to Hardy Weinberg equilibrium (significant if p < 0.05).

Genotypic differentiation test analysis showed that there was no significant difference between the distribution of the L1014F *kdr* allele in suburban area with irrigated agricultural practices (Port-Bouët) and central area without agriculture (Tsassodji) (χ^2^ = 0.00; df = 2; p = 1.00) despite greater tendency of mosquitoes to survive at Tsassodji than Port Bouët in cylinder bioassays. The L1014F *kdr* gene frequencies were found to be in Hardy-Weinberg equilibrium in both natural populations of *An. gambiae s. s.* from Port-Bouët (p = 0.13) and Tsassodji (p = 1.00).

## Discussion

The growing development of insecticide resistance constitutes a serious threat to malaria control programmes and if measures are not taken in time, resistance may compromise control effort in the foreseeable future [4,22]. Monitoring the development of vector resistance in the field prior to the implementation of any malaria vector control initiative is of paramount importance.

This study documented the susceptibility/resistance status of *An. gambiae* to three important pyrethroids commonly used to treat mosquito nets currently distributed across sub Saharan Africa, including Côte d’Ivoire (permethrin, deltamethrin and lambda-cyhalothrin). The focus sites of interest were two distinct areas of Adzopé, a city in Eastern Côte d’Ivoire receiving LLINs donated by the Global Fund initiative. The study was carried out prior to the nets distribution to inform the National Malaria Control Programme of the resistance status of vectors and the need for close surveillance of the resistance phenomenon.

The results presented here show that *An. gambiae s. s.* in the forested areas of Port-Bouët and Tsassodji in Adzopé was exclusively of the M-form. This agrees with a previous study conducted in forest areas of southern Côte d’Ivoire [23]. However, the study was conducted only during May-June and we cannot exclude the occurrence of the S form of *An. gambiae s. s.* during the rest of the year.

Both vector populations have developed various levels of resistance to the three pyrethroids tested. In the bioassays, *An. gambiae s. s.* populations sampled in suburban Port-Bouët area of Adzopé city where more controlled agricultural practices with irrigation system exist, showed higher vulnerability to pyrethroid deposits compared to samples that were collected in the heart of the city (Tsassodji) with no such agricultural practice.

This bioassay observation contrasted with the molecular results: the L1014F *kdr* mutation was detected at both sites but the frequencies of the allele were equivalent (0.67). We do not overlook the implication of the *kdr* mutation in pyrethroid resistance observed in mosquitoes at both sites as this is also supported by the increased in knockdown time (KDT_50_) relative to the normal Kisumu strain. Higher KDT_50_ values in field populations of mosquitoes have been suggested to provide an early indication of the involvement of *kdr* gene in phenotypic resistance [17,24]. However, with same *kdr* rate (67%) but phenotypic difference in expression of resistance by bioassay mortality between both *An. gambiae s. s.* populations suggests the co-existence of both *kdr* and other mechanisms, probably enzyme detoxifiers such as esterases, monooxygenases or GSTs. Over-expression of cytochrome P450 genes associated with pyrethroid resistance is most common in *An. gambiae s. s.*, sometimes in association with the L1014F *kdr* allele [25-28]. No further investigation was conducted to detect additional mechanisms to *kdr* conferring resistance, but one must not preclude any metabolically mediated mechanisms in the pyrethroid resistance observed at Adzopé. Integrated investigations, which allow detection of target sites mutations and metabolic detoxification agents, should be stressed in order to provide a more comprehensive insight into the genetic basis and the mechanisms responsible for the resistance phenotype in these mosquito populations.

The study confirmed the spread of pyrethroid resistance in *An. gambiae s. s.* first detected in Côte d’Ivoire [29], and now in all western African countries investigated [8,30]. This is the first instance of pyrethroid resistance recorded in *An. gambiae s. s.* from the eastern part of Côte d’Ivoire, particularly in Adzopé. This resistance may be explained by the selection pressure from both agricultural and domestic usage of insecticides. Farmers in Port-Bouët admitted to use pyrethroids for crop protection. With agricultural practices, the amount of insecticides being applied to the environment is greatly increasing, and may have a pronounced effect on the mosquito ecology and resistance [31-34]. The protection measures against mosquito bites at both sites in Adzopé are mainly domestic aerosols and mosquito coils. Port-Bouët, the suburban area is densely populated with lower educated classes compared to the higher living standard of people at Tsassodji with modern housing and wealth. The household use of products may explain the resistance level observed at Tsassodji as previously reported in rural Côte d’Ivoire [29].

## Conclusion

Prior to LLINs selective distribution to pregnant women and children <5 yrs in Adzopé, Côte d’Ivoire, resistance to a range of pyrethroids (deltamethrin, permethrin, lambda-cyhalothrin) commonly used to treat these nets was detected at various levels in *An. gambiae*. Mechanisms underlying resistance included high frequency of L1014F *kdr* and other unidentified components, probably metabolic detoxifiers. Their impact on the efficacy of the planned strategy (LLINs) in the area should be investigated alongside careful monitoring of the trend in that resistance over time. The need for alternative insecticides to supplement or replace pyrethroids on nets must be stressed.

## Competing interests

The authors declare that they have no competing interests.

## Authors’ contributions

LPAA and AAK designed the study, conducted the field and laboratory work, the genotyping, interpreted the data and drafted the manuscript. MAA contributed to study design and data analysis. SBA participated in the study design, helped in the mosquito samples collection, the statistical analysis and contributed in drafting the manuscript. PKK participated in the study design and revised the manuscript. RN interpreted the data and revised the manuscript critically for intellectual content. All authors read and approved the final manuscript.
